# Relationship between maternal periodontal disease and low birth weight babies

**Published:** 2013-08

**Authors:** Ahmad Haerian-Ardakani, Zia Eslami, Fahimeh Rashidi-Meibodi, Alireza Haerian, Pantea Dallalnejad, Marjan Shekari, Amir Moein Taghavi, Solmaz Akbari

**Affiliations:** 1 *Department of Periodontology, Faculty of Dentistry, Shahid Sadoughi University of Medical Sciences, Yazd, Iran.*; 2 *Department of Pediatric, Faculty of Medicine, Shahid Sadoughi University of Medical Sciences, Yazd, Iran.*; 3 *Department of Orthodontics, Faculty of Dentistry, Isfahan University of Medical Sciences, Isfahan, Iran. *; 4 *Yazd, Iran.*; 5 *Department of Periodontology, Faculty of Dentistry, Mashahd University of Medical Sciences, Mashhad, Iran.*; 6 *Department of Periodontology, Faculty of Dentistry, Shahed University of Medical Sciences, Tehran, Iran.*

**Keywords:** *Periodontal disease*, *Low birth weight*, *community periodontal indexfortreatmentneeds*

## Abstract

**Background:** Periodontal infections, which serve as a reservoir of inflammatory mediators, may pose a threat to the fetal-placental unit and cause adverse pregnancy outcomes.

**Objective:** The aim of this study was assessing the periodontal status of women during puerperium and determining the possible relationship between their periodontal disease and low birth weight delivery.

**Materials and Methods:** This was a case-control study. The sample included 88 ex-pregnant women were seen at maternity hospitals of Yazd, Iran. Half of the mothers had low birth babies (LBW) (birth weight below 2500g- case group) and the others had normal weight babies (>2500g- control group). The mothers’ data were obtained from medical files, interview and periodontal clinical examination carried out up to 3 days after delivery. Bleeding on probing, presence of supra-gingival calculus and CPITN (Community Periodontal Index for Treatment Needs) were used for periodontal assessment

**Results:** Among the known risk factors of LBW babies, history of previous LBW infant among case mothers reached statistical significance (p=0.0081, Student *t*-test). Mothers of LBW infants had less healthy areas of gingiva (p=0.042), and more deep pockets (p=0.0006, Mann-Whitney test).

**Conclusion:** The maternal periodontal disease can be a potential independent risk factor for LBW.

## Introduction

Maternal health conditions associated with chronic decrease in uteroplacental blood flow (maternal vascular diseases, preeclampsia, hypertension, maternal smoking) are associated with poor fetal growth and nutrition (1). Low birth weight (LBW) babies, defined as babies having birth weights of less than 2500g, represented disproportionately large component of neonatal and infant mortality rates. Although LBW babies make up only about 6-7% of all births, they account for more than 70% of neonatal deaths (2). Infections may play an important role in prematurity (3, 4). The primary mechanism is ascending infections from the vagina, which is associated to 50% of preterm birth. Other infections remote from fetalplacental unity were also regarded as a potential risk factor for preterm birth (4). 

Periodontal disease is one of the common conditions that is responsible for a chronic inflammatory challenge in the body. This group of diseases happens in consequence of organized biofilm present on tooth surfaces. The microbial biofilm releases substances that activate the immunoinflammatory responses of the host (5). This challenge could trigger inflammatory mechanisms associated with preterm birth outcomes (6). Recently, periodontal disease was known as a risk factor for preterm birth or low birth weight (PLBW) because the bacterial migration from periodontal tissues into blood circulation may stimulate the production of inflammatory mediators responsible for the onset of delivery (7-13). 

The focal infection theory proposed by Hunter in 1910 was being resurrected. According to Hunter’s theory, bacteria and their products from local infections could be disseminated throughout the body and cause diseases in other organs and remote infections such as periodontitis and premature birth can be linked, as the microbes themselves or microbial toxins entering the uterine cavity during pregnancy by the ascending route from the lower genital tract or the blood borne route from a non-genital focus (14). Microbes or their products then interact, most likely in the decidua or possibly in the membranes, leading to prostaglandins production or directly to uterine muscle contraction. This interaction is mediated through a cytokine cascade (15). Inflammatory periodontal tissues release significant amounts of pre-inflammatory mediators mostly interleukin 1β, prostaglandin E_2_ and TNFα which have several systematic effects on the host (16, 17). 

Most studies have evaluated the relation between periodontal disease and PLBW, but because preterm birth is a low weight risk factor itself, in this study the relation between periodontal diseases and full term LBW infants is assessed.

## Materials and methods

88 ex-pregnant women which had attended to Gynecology Department of Hospitals and birth centers in Yazd for delivery from 2009-2010, with no systemic problems before or during pregnancy recorded in their medical history were chosen and allocated into two equal groups. The case group was consisted of 44 women who gave birth to infants weighing less than 2500 gr, and the control group gave birth to infants weighing more than 2500 gr. The mean age of both groups was 24 years. Exclusion criteria were as followed:

1. Pregnancy period less than 37 weeks.
2. Women who needed antibiotic prophylaxis before dentistry services.
3. Women with a history of previous uro-genital infection who had received antibiotic therapy.
4. History of systemic diseases such as diabetes, heart disease, glumeronephrites and maternal hyper-thyroidism.
5. Mothers who gave birth to twins.
6. Smokers or alcohol consumers.

After assessing the dossiers, samples were chosen from these hospitals: Shohadaie karegar, Mojibian, Mother, Goodarz, Bahman in Yazd. Each case had a similar counterpart in the control group (Individual Matching). Maternal age was the criteria for choosing the control group. Women were examined within 3 days after labor by a trained examiner, blind to the groups. 

All participants had signed the testimonial. Questionnaire or the patient dossier gathered this information: Sex and weight of the infant at birth, maternal age, starting point of pre partum cares, number of checkups during pregnancy, history of scaling in the recent pregnancy period. Periodontal assessment was carried out using a UNC15 probe and a mirror, which included the following recordings:

- Supra gingival calculus, presence or absence of any calculus detected by probe sounding or observation.
- Bleeding on probing, presence or absence, following gentle probing around teeth.
- CPITN (an index aimed for assessing the need for periodontal treatment).

The dentition is divided into six sextants (one anterior and two posterior tooth regions in each dental arch). The periodontal conditions are scored as follows:

- Grade 0 is given to a sextant with no sign of pocket, calculus and bleeding on probing (gingival health with no treatment needs).
- Grade 1 is given to a sextant with no pockets, calculus or overhangs of fillings but in which bleeding occurs after gentle probing in one or several gingival units (mild gingivitis; improving oral hygiene is needed).
- Grade 2 is assigned to a sextant if there are no pockets exceeding 3 rnm, but in which dental calculus and plaque-retaining factors are seen or recognized subgingivally (established gingivitis; scaling, removal of overhangs, and improved oral hygiene is needed).
- Grade 3 is given to a sextant that harbors 4-5 mm deep pockets (mild periodontitis; scaling, removal of overhangs, and improved oral hygiene is needed).
- Grade 4 is given to a sextant that harbors pockets 6 mm deep or deeper (periodontitis and complex treatments needed).

This study was approved by ethical committee of Shahid Sadoughi University of Medical Sciences, Yazd, Iran before beginning.

**Statistical analysis**

Statistical analysis was done with SPSS 10 software. The mean differences of CPITN data were analyzed with Mann-Whitney non-parametric test with a significance level of p≤0.05. Analysis of periodontal indices data was performed with independent student’s* t* test (p≤0.05). Wherever there was non-continuous data, Chi-square test was used.

## Results

**Figure 1:**
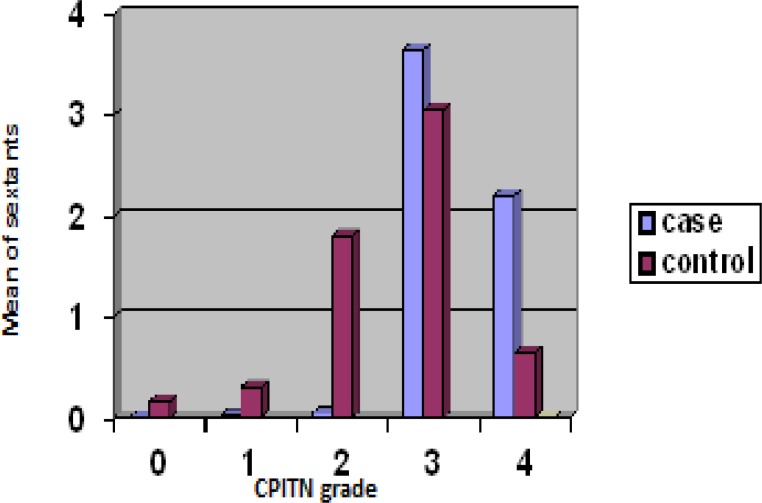
Comparison of sextants with different CPITN grades between case and control groups

This case-control study was performed on 88 women equally divided into two groups. 4900 sites were examined in the case group and 5168 sites in the control group. The mean number of sextants with CPITN grade IV (or periodontitis) was significantly higher in the case group (p=0.0006 Mann-Whitney test) but the mean number of sextants with CPITN grade zero or healthy gingiva (p=0.042), grade I or mild gingivitis (p=0.002), grade II or established gingivitis (p<0.0001) was significantly higher in the control group (Figure 1). 

Percentage of the sextants diagnosed with periodontitis (CPITN grade III and IV) in women with LBW infants (case group) was 1.6 times more than the control group. The number of sites that had bleeding on probing was significantly higher in the case group (p<0.0001, student’s *t* test). The amount of supragingival calculus was also significantly higher in the case group (p=0.007, student’s t test). Among the LBW risk factors, only previous history of LBW babies was significantly higher in the case group (p=0.0081). There was no significant difference between the two groups in other risk factors: husbands’ job, infant’s sex and mother’s educational level (p=0.068 Chi-square test). Maternal weight gain was significantly higher in the control group (p=0.035 Chi-square).

## Discussion

This study showed that mothers with periodontal disease relatively gave birth to low-weight babies. The relationship between periodontal disease and LBW babies was assessed and women with preterm babies were excluded because, being preterm is a major LBW factor. Other studies have mostly assessed the relation between periodontal disease and low-weight, due to short pregnancy period. Therefore this study is more reliable in showing periodontal disease as an etiologic factor for LBW independent from pregnancy period length. 

Periodontal tissue destruction has a cumulative age-related effect; therefor the case and control groups were individually matched. The mean number of sextants with CPITN grade zero, I, II was significantly higher in the control group; but the mean number of grade IV sextants (or periodontitis) was significantly higher in the case group (p=0.0006); which means periodontal health in case group was poorer than control group.

Dasanayake *et al* reported that the number of sextants with CPITN grades I and II was higher in the case group (18). Sextants with grades III (shallow pockets) and grade IV (deep pockets) were also higher in the case group but the difference wasn’t significant. It appears that the low number of grade IV sextants was the reason that the difference wasn’t significant. In this study the numbers of grade IV sextants was significantly different between the case and control groups, which were similar to other studies (17, 19-22).

Khadem *et al* in a similar study to ours , but in different city (Mashhad, Iran), showed that percentage of sites with more than 3 mm in probing was significantly higher in case group (21). Alves and Riberio reported an obvious relation between periodontal disease and PLBW with an 8/9 odds ratio (22). Jeffcot *et al* reported similar results and stated that the risk of having a LBW or preterm infant increases 4-7 times depending on the severity of the periodontal disease (19). Another study by Lopez *et al* about pregnancies with gingivitis confirms these results (23). Santos-Pereira *et al* studied on 124 Brazilian women and showed a correlation between chronic periodontitis and LBW/preterm birth (24). 

The result of a meta-analysis study by Chambrone *et al* showed a significant risk of preterm delivery for pregnant women with periodontitis (risk ratio (RR):1.70) and a significant risk for LBW (RR: 2.11) (25). Corbella *et al* have been made a review based on case-control studies to evaluate role of periodontal disease as a risk factor for preterm birth, low birth-weight babies (26). The estimated odds ratio was 1.78 for preterm birth, 1.82 for low birth-weight and 3.00 for preterm low birth-weight in mothers with periodontal disease. But, despite the results of the analysis of pooled data in these 2 systematic reviews which suggested a link between periodontal diseases and adverse pregnancy outcomes, a high and unexplained degree of heterogeneity between studies was mentioned by the authors.

On the contrary Mitchell-Lewis *et al* reported that periodontal disease had no significant effect on having full term or preterm infants, although the authors stated that periodontal therapy before labor decreases PLBW by 28% (27). Lunardelli and Peres found no relation between maternal periodontal disease and LBW but there was a relation between periodontal pockets and preterm babies, which was affected by other maternal health variables (20). Noak *et al *using a logistic regression model, showed that periodontitis is a risk factor for PLBW. But among the risk factors related to pregnancy that were evaluated, only previous history of LBW was statistically significant (p<0.0001, t-test) (28). 

According to this study results, it can be concluded that maternal periodontal disease could be an independent risk factor for LBW babies. Preterm delivery and low birth weight may lead to infant mortality, high treatment expenses, and the consequent emotional problems for family specially mothers; so it is suggested that periodontal assessment before pregnancy and during this period must be considered as a part of health care protocols for future mothers 

## Conflict of interest

The authors have no conflict of interest.
